# Enhancing energy literacy in children using zn/cu/potato batteries

**DOI:** 10.12688/f1000research.13228.1

**Published:** 2018-01-08

**Authors:** Mark Polikovsky, Avigdor Sharon, Alexander Golberg

**Affiliations:** 1 Porter School of Environmental Studies, Tel Aviv University, Tel Aviv, Israel

**Keywords:** energy education, batteries, lighting, household air pollution prevention, bioelectricity

## Abstract

**Background** **.** The major challenges that prevent the wide-scale adoption of emerging personal clean energy production are unawareness and low self-confidence. We tested a hypothesis that a combination of a new technology and educational methods could lead to the increase in awareness of children to clean energy possibilities and to an increase in self-confidence in applying them.

**Methods** **.** Here we report on a toolkit that combines low carbon, clean energy source, Zn/Cu/potato batteries, sufficient to power light-emitting diodes, with a non-formal education by experience program, based on case studies and hands-on experience with battery assembly for 6-11 years old children, led by trained 12-14 old youth leaders.

**Results** **.** The results show that the education experience increased the awareness of the children to produce electricity at home from unconventional, yet available raw materials and their self-confidence in being able to do this (p=0.008).

**Conclusions.** The developed toolkit supports environmental and energy literacy education through non-formal training, increasing awareness and self-confidence in children to actually apply this in their living environment to produce clean energy.

## Introduction

Children are the decision-makers of the future and will bear the brunt of the energy-environment- climate crisis ^1– 3^. The USA Department of Energy Literacy guide stays:

*Without a basic understanding of energy, energy sources, generation, use and conservation strategies, individuals and communities cannot make informed decisions on topics ranging from smart energy use at home and consumer choices to national and international energy policy* ^4^.

A study of USA youth ^5^ revealed that “students did not have a sound knowledge and understanding of basic scientific energy resources facts, issues related to energy sources and resources, general trends in the U.S. energy resource supply and use. The students also did not understand the impact of energy resource development and use on society and the environment” ^5^. Similar results were observed in recent studies with New Zealand ^6^ and Taiwanese ^7^ youth, who mostly looked at energy in monetary terms and knew little of its environmental impacts. A UK study showed “that children derived more motivation to save energy from responsibility conferred by school activities than other (e.g. environmental) concerns, and some connected energy saving with dangers of using electricity (e.g. fire)” ^8^.

Most children learn about energy issues from parents ^6, 8^. Therefore, the energy literacy of young persons in low income countries is lower than in OECD countries because of the general population's unawareness of renewable energy potential and of energy impacts on health and the environment ^9^. Developing education tools to increase awareness to the effect of energy production on health and environmental problems is a major challenge, required to make a change in this sector.

Our long-term goal is to increase energy literacy among children by creating awareness to the hazards of toxic and polluting materials, the benefits of clean energy, and the possibilities of creating clean energy at home. Subsequently, to encourage learning and actual implementation, resulting in a behavior shift towards using clean energy on an on-going basis.

**Figure 1.**
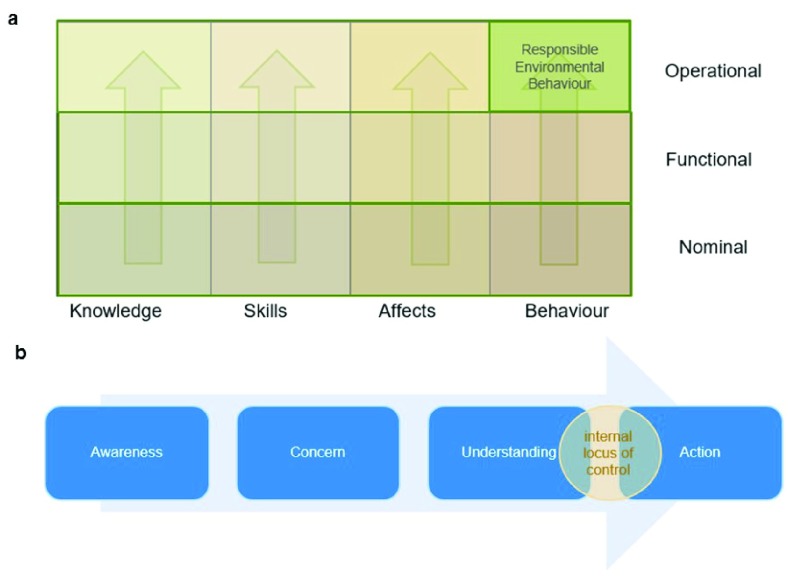
Developing Environmental and Energy-responsible literacy. ( **a**) From rudimentary knowledge and budding awareness to responsible environmental behavior: the environmental literacy continuum. ( **b**) The stages of developing environmental and energy-responsible literacy in education.

To achieve this goal it is important to provide not only the required knowledge, but also to develop interest, care and a perception of personal ability. These are all attributes of environmental literacy. Environmental literacy is the ability to understand the environmental systems that we live in, their importance and impact on us, as well as our own well-being requirements and to take the measures required to interact with the environment in a responsible fashion and to benefit from it while sustaining it for the long term ^10^. It requires both cognitive and affective elements, encompassing knowledge, affects, skills and behavior, which are all important to develop responsible environmental behavior ^10– 12^. As people develop their environmental literacy, they progress gradually from a basic, nominal level of their knowledge, emotions, skills and behavior to functional and operational levels ( Figure 1a). Though each individual has a unique journey through the continuum of environmental literacy development, it is usual to go from initial awareness to developing personal concern and then better understanding and a perception of personal ability before deciding to take action ^10^ ( Figure 1b).

Gaining knowledge does not necessarily lead to developing pro-environmental attitude, care and values. Developing attitudes does not necessarily lead to taking action and to adopting environmentally responsible behavior ^13, 14^. Some triggers or specific ingredients of the learning experience and of life experience as a whole seem to be required to develop responsible environmental and energy-wise behavior. Such ingredients are personal experience and emotions, curiosity, sense of wonder and excitement ^15– 20^

Previous works suggested various scenarios to develop energy literacy through a standard curriculum in classes ^21^. An alternative approach is an outdoor, non-formal education. Indeed, outdoor education, in a non-formal setting, was found to result in better behavioral outcomes in comparison to traditional classroom learning ^22^. Several important attributes such as concentration, agility, emotional expressions, and communication were found to be higher in the outdoor education setting. It was reported that “Outdoor education influenced behavioral changes in a positive direction” ^22^. Non-formal education at various settings provides new opportunities for learning and benefits for both the effective and cognitive axis of human behavior, inciting interest, positive attitudes and fond memories that persist over time ^23^. The non-formal education, in an outdoors or community settings, is beneficial and synergistic with both school learning and family engagement ^24^. Therefore, it is of an important significance in developing environmental and energy literacy and in nurturing responsible energy and environmental behavior in communities and families. Consequently, novel tools are needed to nurture this behavior.

More than two centuries ago, Luigi Galvani’s pioneering research of the electrical properties of biological tissues discovered “bioelectricity”. Inspired by those results, Alessandro Volta invented a device capable of producing electricity by the mere contact of conducting substances of different species” ^25^. This device, the ‘Voltaic battery’, marked the birth of a new era in the development of modern physics and important changes in our lifestyle by using batteries. The key advantage of batteries is their ability to store and deliver portable, off-the-grid electricity.

In the application for lighting in low-income off-grid communities, batteries already allow for the transition from fuel to electric-based lighting and could reduce health risks ^26^. In addition, by supporting the elimination of fuel-based light sources, they would also contribute to the reduction in greenhouse gas emissions ^27^, weaning governments of the burden of fuel subsidies that often exceed expenditures on healthcare and, importantly, provide new jobs ^28, 29^. However, improperly disposed commercial batteries contaminate the environment with heavy metals such as mercury, lead, cadmium, and nickel. In addition, battery energy is the key cost component in all low-cost devices recently developed for low-income communities ^30– 33^.

**Figure 2.**
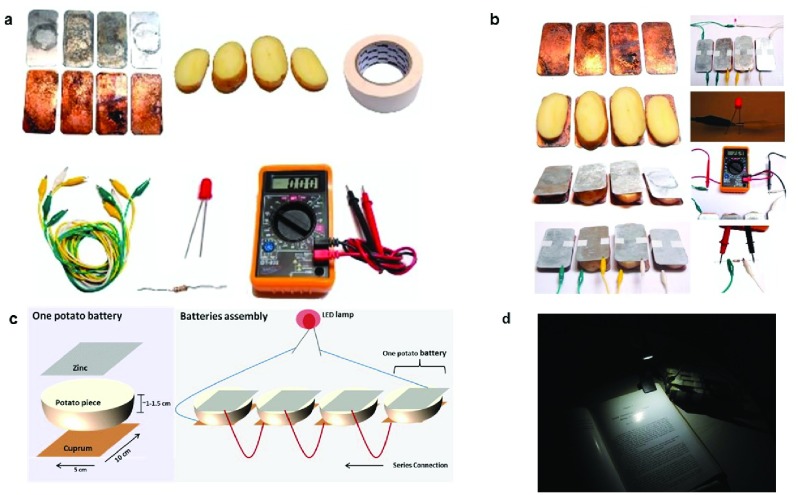
“Make yourself a battery” toolkit. ( **a**) Basic components required to assemble a battery at home. Zinc and Copper plates (~5×10cm), wires, boiled potato, sealing tape, light emitting diode (LED). Test resistor and voltmeter are used for performance measurements. ( **b**) Process for a single cell and complete battery assembly. Assembly description of four potato batteries to turn on one LED lamp ( **i**) put 4 plates on a rigid substrate; ( **ii**) add 4 boiled slices of potato, once cooled, on the metal plates, recommended to cover the metal plates with the potatoes, as much as possible; ( **iii**) cover the potatoes with the Zn plates; ( **iv**) seal the cells with a tape and connect the batteries with the cables - each metal should be connected to the closest battery to the second metal except to metal plates on the edges; ( **v**) connect the not connected plates, to the LED lamp, the electric circle is closed; ( **vi**) in a case everything connected right, the LED lamp will be turned on; ( **vii**) to measure open circuit voltage, a voltmeter should be connected instead the lamp; ( **viii**) to measure the current in the system, connect the 100ohm resistor instead of the LED and measure the voltage on it. ( **c**) Schematic representation of a complete lighting device, powered by Zn/Cu/boiled potato battery. ( **d**) Lighting device performance example for book page illumination in a completely dark room.

To address the problems of availability, cost and environmental impact, we introduced vegetative batteries, based on Zn/Cu and potatoes with disintegrated cell walls. Preliminary cost analysis showed that a Zn/Cu-Potato battery is able to produce portable energy at ~7$/kWh, which is significantly lower than current available 1.5 Volt AA alkaline cell (~450$/kWh (retail)), flow (~600$/kwh) ^34^, advanced lead-acid (900$/kWh) ^34^, lithium ion (500$/kWh) ^34^ or D cells (~49-84$/kWh) ^35^. The basic battery cell design and resulting LED light ( Figure 2) allows for reading a book in a completely dark room ( Figure 2d). In comparison to kerosene lamps with lighting efficiency of 0.08-0.11 lm/watt, light-emitting diode (LED)-Zn/Cu-boiled potato battery provides 8.3-53.1 lm/watt. At the same time, cost of light is 0.13-0.85$/1000lm∙h, while for kerosene lamps it is 3.69-5.81 $/1000lm∙h ^35^. This study was followed by a paper from Sri Lanka, which reported 500 hours operation LED with Zn/boiled plantain pith battery ^36^. Yet, the rate of adaptation of new technologies in population is low. Low awareness and low self-confidence contribute to these low rates of technology adaptation ^37^.

The goal of this work is to test the working hypothesis that Zn/Cu-boiled potato battery technology, taught using participatory outdoors educational methods, could lead to the increased awareness and self-confidence of young children in actually producing clean energy at home.

## Methods

### Study subjects

96 children aged 6–11 years and 11 youth guides 12–14 years old from Israel participated in the study in the “Teva HaSviva” summer camp in July-August 2015. Written informed consent was obtained from parents before the camp for their children’s participation in the study as part of their camp activities, according to the Israel law, by “Teva HaSviva”. The parents and the children were informed that the camp runs experimental educational programs in various branches of environmental education.

### Zn/Cu/potato battery assembly

We developed an easy to use toolkit for light generation from LEDs. The important aspect of the potato battery toolkit ( Figure 2a) is that it can be readily assembled at any home or any outdoor activity in the majority of the countries in the World. The basic elements of the battery are Zn and Cu electrodes, wires, LED, knife, boiled potatoes ( Figure 2a). The steps for battery preparation, assembly, and testing appear in Figure 2b. Although in these studies we used conventional boiling, used by families to cook, alternative cleaner energy strategies, such as solar cooker can be used ^38^.

In this work, we developed a “Make yourself a battery” toolkit to teach youth energy and environment issues with the potential to provide light and prevent household poisoning. We demonstrated the use of the kit as an educational tool in a pilot study with 96 children 6–11 years old. This kit can be used as an energy and environment educational technology for the outdoor educational activities. In addition, it can displace the kerosene lamp for reading in medium and low- income countries. The kit provides a low-cost technology that can serve the dual purposes of improving lighting efficiency and increasing environmental and energy literacy by removing unawareness and low self-confidence, which are major barriers for the introduction of new energy systems to low-income countries.

During the activity, the children used boiled potatoes, Zn and Cu electrodes (5X10 cm), wires, resistors, voltmeter and LEDs, as described Figure 2a. The assembly protocol for each cell and the complete battery, which consists of several cells connected in series, is shown in Figure 2b and c. Figure 2 shows a step-by-step procedure for battery assembly and use to generate LED light sufficient for reading ( Figure 2d)

### Zn/Cu/potato battery performance evaluation

The thickness of each galvanic cell was measured by ruler (1mm resolution). The working surface area was measured in digital images of each galvanic cell using ImageJ (ver 1.48r 18) program (NIH, ML). Open circuit voltage and the voltage on the 100ohm resistor were measured using a digital voltmeter (0.01 V resolution). Current flowing on the resistor was calculated by Ohm’s law as I=V/R. Power was calculated as P=I∙V. The current and power density were calculated by equations *i*=I/A ( *i*=current density, A= area in cm ^2^) and p=P/A, respectively ^39^.

### Pilot study design: Educational program to provide early age education for generating clean electricity for light

To increase energy literacy and the use of the “Make yourself a battery” toolkit, we designed the following pilot study. First, we further developed the technology of batteries based on Zn and boiled potatoes ^35^, which can be easily used in low-income communities. Second, we assessed the pre-learning energy literacy of a group of children. Third, we performed a hands-on activity with the children who assembled and characterized the Zn/boiled potato batteries. Finally, we assessed the post-learning energy literacy of this group of children.

To enhance the environmental and energy literacy in youth we developed an outdoor activity that can teach children about energy conversion and light generation. The study was conducted at the “Teva Hasviva” summer camp at Tel-Aviv University, Israel in 2015. The camp takes place during every school vacation period that is at least 1 week long, throughout the year. The core themes of “Teva Hasviva” camps are sustainable living, care for nature and ecological literacy. The core mission of the camps is to foster environmental literacy, synergy with nature in and around the city, nature conservation and a healthy lifestyle. This is done in an enjoyable setting, learning by experience in a combined outdoors and university campus environment, having fun, leading activities with a practical contribution to the natural environment and to the community, research and discovery.

**Figure 3.**
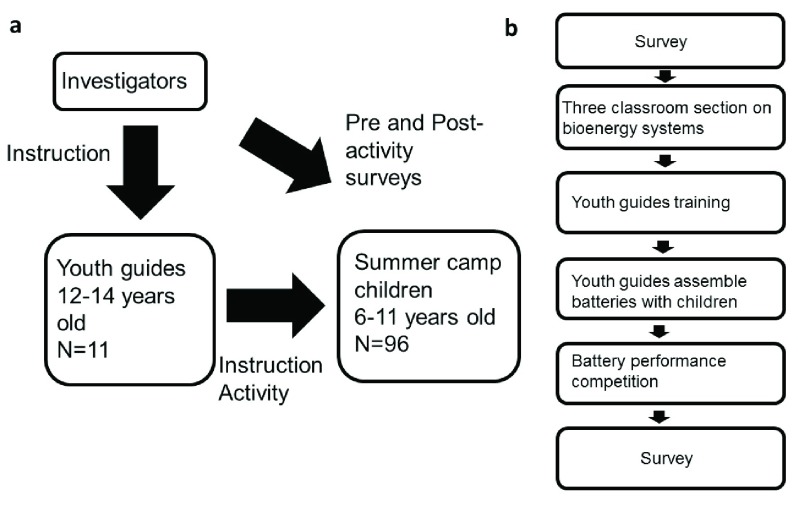
Educational activity description. ( **a**) The schematic description of the educational activity involving youth leader and children in the summer camp. ( **b**) Teaching materials, lectures, and activity structure as performed during the camp.

The study design is shown in Figure 3a. The summer activity is also a part of a 3-year qualification program for youth guides. For this study, we first trained young youth guides (12–14 years old), participating in a course during the same summer camp (N=11). The trained youth guides performed all the activities with children (6–11 years old, N=96), who participated in a 2-week summer camp. Additional activities for participating children included visiting natural open spaces near the city and conducting animals and plants surveys, building nesting boxes for birds, making creative artifacts from packaging materials to avoid dumping, learning to make healthy, natural food, plant ecological gardens, in a learning-by-doing approach ( Supplementary File 1 and Figure 3b). Each part of the activities took 30–40 min.

The targets of the education activity were defined as follows:

1. Theoretical introduction to energy in nature and society.
2. Youth guides will learn how to teach and how to perform an experiment and then perform the experiment with the children.
3. Children will learn about concepts of energy generation and its application to affluent and low-income countries problems.
4. Children will learn of the actual production of electricity from Zn/Cu/potato batteries as an educational activity and as a tool for them to make their own battery to use.
5. Development of energy education tool and protocol for rapid batteries assemblies for low- income remote communities.

Youth guides, who participate in the three-year environmental leadership program of the camp, were instructed before their activity on the method to assemble the battery and measure the outputs of produced electricity. They practiced the theory of what they learned during the theoretical presentation. Moreover, they learned how to use the voltmeter to measure voltage and current and measure effective electrolyte surface area by digital photography. Each of the youth guides led a group of 5–8 children during the practical battery assembly activity. The assembly was done in a form of competition between groups. Youth guides taught the children about electricity production from batteries for lighting and showed them how to use the voltmeter. The winner was the team with maximum generated voltage. Youth guides were also responsible to collect measurements from the assembled batteries such as electrolyte area, number of connected-in-series cells, total voltage and current ( Table S1 and Table S2). 

In addition, to demonstrate to children that it is possible to provide human needs without damaging the environment, the complete life cycle of potatoes used in the process was shown ( Figure S1). After the activity, all used potatoes were composted. No leftovers were thrown away. We explained that the composted potatoes could be used for fertilization. We explained that the metal plates, cables, and LED lamps could be reused for additional activates. This way we introduced the entire cradle-to-cradle lifecycle of all materials used. We described a process where we minimize any use of materials that could not be naturally recycled. In places where the teaching period is longer than a few weeks (as was in our case), and the places include area for potato growth and compost it can be useful to show the children the potato lifecycle before and after the activity, including cultivation of new potatoes using the compost from the previous ones. This activity can also be integrated with a field implementation of solar cookers ^38^.

### Learning process evaluation

A short pre-learning questionnaire and a matching short post-learning questionnaire were used. In both questionnaires, Likert-type questions (a 1–5 scale) for quantitative assessment of levels of knowledge, skills and attitudes and open text questions for qualitative analysis were used ( Table 1, see Supplementary File 2 for detailed questionnaire structure). The questions addressed the major areas of environmental and energy literacy continuum with an emphasis on affects as a key to literacy development. Pre-learning questions were designed to find out the extent of awareness and the level of knowledge before the learning session took place. Post-learning questions were designed to find out what participants have learned and understood, what they remember and what was their attitude towards the information and insights from the learning session. All 96 children participating in the summer camp received the pre-learning questionnaire 4 days before the learning session. They received the post-learning questionnaire 4 days after the learning session. Both questionnaires were provided in a very casual, relaxed atmosphere during a time break at the orchard area where the children had lunch and rested. The questionnaire was kept short to make it easy for the children to reply to it despite distractions in the field. The results were then analyzed and compared.

**Table 1.** Pre- and post-learning activity questions, addressing the major elements of environmental literacy.

	Pre	Post
Knowledge	Electricity is always available. It never runs out	Electricity is always available. It never runs out
	It is possible to create electricity on our own	It is possible to create electricity on our own
	Which ways do we get or create electricity	Which ways do we get or create electricity
	How can we create electricity on our own	How can we create electricity on our own
	Can there be interruption in our electricity supply	--
	How does a battery work	How does a potato battery work
	--	How can we create battery from potatoes
Skills	I can create electricity by myself at home	I can create electricity by myself at home
Affects (awareness, interest, attitudes)	Nature has cool things that are interesting to know about	Nature has cool things that are interesting to know about
	We can learn from nature very useful things for us	We can learn from nature very useful things for us
	I think it will be interesting for me to learn about energy in nature	It was interesting for me to learn about energy in nature
	I think it will be interesting for me to learn to create electricity from potatoes	It was interesting for me to learn to create electricity from potatoes
	I am curious to see what we will learn	I enjoyed our learning session
Self-locus of control	--	I can teach others what we learned
	--	Can we create enough energy for a number of lamps? Do we need to get help from others to do it?

### Statistical analysis

In addition to descriptive statistics for the Likert-scale questions, Pearson correlation was computed to assess the relationship between answers to questions in the Likert-type questionnaire. Correlations are reported in APA style with the syntax r= Pearson’s correlation value, p=significance value. Student’s t-test (2-tailed, DF=is shown for each t-test result) was used to examine differences in variables before and after the learning session. All analysis was done with IBM SPSS Statistics ver 23 (IBM, NY).

Some questions before and after the learning session were not exactly the same, therefore, no T value was calculated (before: “I think it will be interesting for me to learn about energy in nature”, after: “It was interesting for me to learn about energy in nature”).

## Results and discussion

### The electricity production from boiled potato batteries with Zn/Cu electrodes

**Figure 4.**
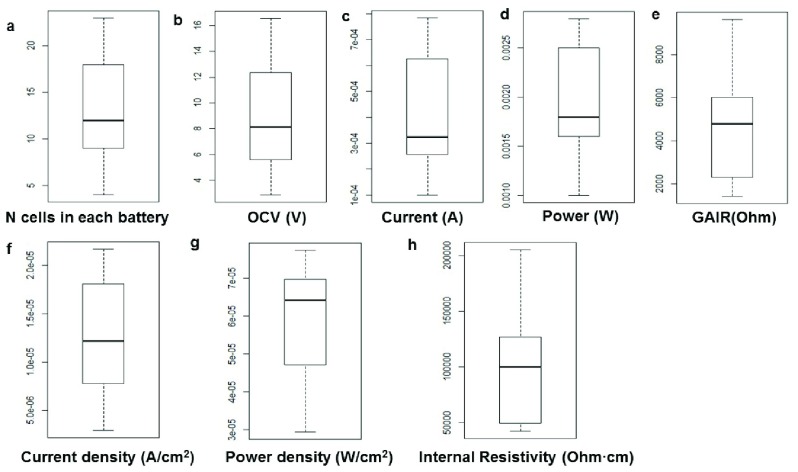
Distribution of assembled in competition batteries performance. ( **a**) Number of cells in the final battery. ( **b**) Open circuit voltage (OCV). ( **c**) Current. ( **d**) Power. ( **e**) Galvanic apparent internal resistance (GAIR), which show how much energy is lost by battery internal resistance. ( **f**) Current density. ( **g**) Power density. ( **h**) Internal resistivity.

The generated voltage, current, power, current density and power density from the assembled batteries are shown in Figure 4. The longest assembled by children battery consisted of 23 individual cells ( Figure 4a). The largest generated open circuit voltage was 16.55 Volt ( Figure 4b). The largest current was 2.8mA ( Figure 4c), the largest generated power was 0.78mW ( Figure 4d). The lowest battery galvanic apparent internal resistance was 1,424 Ohm ( Figure 4e). The maximum generated current density was 77 µA cm ^-2^ ( Figure 4f) power density was 22 µW cm ^-2^ ( Figure 4g). The lowest galvanic internal resistivity was 42,320 Ohm∙cm ( Figure 4h).

### Pre-learning attitude, awareness, and interest

A short pre-learning questionnaire and a matching short post-learning questionnaire were used ( Table 1. See *Methods*). In the pre-learning questionnaire, the children demonstrated a very high level of interest to learn about nature (4.38±0.92, on Likert-type 1–5 scale), as may be expected for children participating in a nature and environment summer camp. They also expressed a very high level of agreement that nature holds practical and useful value for us. This insight and the interest to learn were correlated (r=0.406, p=0.0).

The children were looking forward with much interest to learn and experience the creation of electricity from potatoes (4.23±1.23, on Likert-type 1–5 scale); less interest was expressed to learn about energy in nature in general (3.98±0.84). Although agreeing that we can create electricity by ourselves, they showed only an average level of confidence that they can do it. This infers a low confidence in their skills and locus of self-control. The interest to learn about creating electricity from potatoes was correlated to the interest to learn about energy in nature (r=0.398, p=0.0), but no correlation was found to the concept of creating electricity at home or to the level of confidence that they can do it ( Table S3). On the other hand, there was a positive correlation between the perception that it is possible to create electricity on our own and the confidence that they can actually do it although they had no experience yet (r=0.375, p=0.0). Both findings show a positive interest and anticipation before the learning experience but little awareness and self-confidence in actually putting it to any practical use. We concluded that though they looked forward with positive anticipation to the learning session, they generally tended to expect it to be a curiosity with no practical implementation of actually producing electricity at home. Therefore, a major challenge for the learning activity for these children was to demonstrate and understand the practical implementation possibilities and to develop a perception of self-confidence in their capabilities to implement it. If the learning process would succeed with children in an affluent society in which power from the grid is constant and commonplace, it could potentially succeed even more in communities with no power from the grid. For future studies, we suggest to repeat this study with children in countries where power from the grid is scarce or non-existent and compare their attitude to the same process. It could be expected that they would be more aware from the start to the practical implementation yet this should be tested in a study.

### Post-learning impact

Comparing the results before and after the learning session ( Table 2 and Table 3), there was one significant difference - the perception that “I can create electricity at my home”. Since appreciation and interest to learn about nature was rather high to start with, a change in attitude was not expected to be significant. The challenge was in acquiring the skills and self-confidence or locus of control. A Student’s t-test analysis shows that there was a significant change in the perception that children can create electricity at home by themselves with such simple ingredients such as potatoes (t(178) = -1.74, p = 0.008), with higher scores after the learning session.

**Table 2.** Comparison of knowledge before and after the learning session. “Which ways do we get or create electricity” top 7 replies.

Pre			Post		
Reply	N	%	Reply	N	%
From potatoes	39	41	From potatoes	44	52
Electric Company / power stations	20	21	Sun, Solar energy	15	18
Sun, Solar energy	13	14	Electric Company / power stations	13	15
Don’t know	11	11	declined to answer	10	12
declined to answer	9	9	Don’t know	6	7
Static elec. (comb, balloon)	7	7	Coal	3	4
Batteries	6	6	Water	2	2

**Table 3.** Comparison of knowledge before and after the learning session. “How can we create electricity on our own”.

Pre			Post		
Reply	N	%	Reply	N	%
Just stating “From potatoes”	39	41	Just stating “From potatoes”	51	60
Don’t know	13	14	Declined to answer	10	12
Declined to answer	10	10	From potatoes – explaining exactly how	10	12
Sun, Solar energy	6	6	Don’t know	8	9
From potatoes – explaining exactly how	5	5	Sun, Solar energy	7	8
Static elec. (comb, balloon)	5	5	Vinegar	2	2
Batteries	3	3	Vegetables	2	2

There was a marked correlation between the children’s perception that they can create electricity at their home and their thoughts that it was interesting for them to learn about energy in nature (r=0.328, p=0.02), interesting to learn to create electricity from potatoes (r=0.390, p=0.0) and that they enjoyed the learning session (r=0.430, p=0.0), Table S3.

Children that thought they could create electricity at home, were more comfortable to feel they can teach others what they learned (r=0.375, p=0.0). Again, so did children that thought it was interesting for them to learn about energy in nature (r=0.443, p=0.0), interesting to learn to create electricity from potatoes (r=0.617, p=0.0) and that they enjoyed the learning session (r=0.636, p=0.0). There was a high correlation between these insights ( Table S3). These results show that achieving engagement and interest in the learning activity has a positive effect on developing perceptions of personal capabilities to actually create electricity at home from potatoes and to teach others.

These results for a learning session in the midst of an intensive summer camp show the potential of hands-on learning experience to set understanding and awareness and most of all, an appreciation of the personal capability to do it and make a change.

### Qualitative assessment of knowledge and attitude

The purpose of the qualitative (open text) questions before the session was to find out the extent of awareness and the level of knowledge before the learning session took place ( Table 2 and Table 3). The purpose of the questions after the session was to find out what they have learned and understood, what they remember and what was their attitude towards the information and insights from the learning session.

When asked before the learning session: “How do we get or create electricity?” only 24% of participants mentioned the electric company or power stations, 14% mentioned solar energy, 20% either declined to answer or said they did not know, 13% talked about static electricity that they probably learned about in school or about batteries. Additional children mentioned non-relevant answers. Yet, the big surprise was that a remarkable 41% mentioned potato based battery as a source of electricity. It might be assumed that as they knew the questionnaire is related to the learning session, they thought that that was the reply we expected or wrote it for lack of any other thought.

In the post questionnaire, which was provided on the week following the activity, there was a marked difference in replies to the same questions. When asked this time how do we get or create electricity, 23% mentioned solar energy and other renewable energy sources. Potatoes reined high with 52% of replies. Only 19% mentioned the electric company or power stations, and 15% declined to answer or said they did not know. These results show that a week after the learning session there was a high acclamation to the use of renewable energy sources for electricity and of the possible use of potato batteries as was experienced at the learning session.

Consequently, it appears that before the learning activity only 33% of the children mentioned valid sources of electricity at home for daily use. After the activity, there was a larger awareness to solar energy and other renewable sources. Non-relevant sources, such as lightning and static energy, that appeared on replies before the learning activity (13% of the children) were dropped after the activity.

When asked what they know about the possibility of creating electricity at home, 60% mentioned again potatoes, but an additional 12% knew to explain exactly how it is done. Another 5% talked about using lemon or vinegar and 8% talked about renewable energy sources. 24% either declined to answer or said they did not know, 8% talked about static electricity or batteries, and 3% said explicitly that it could not be done.

Before the learning session, 18% of participants knew about ways to create electricity at home, 23% had no idea, and 41% just wrote “potatoes”. After the session, 15% of the children provided a detailed account of how to make a battery while an additional 18% provided a partial explanation. Additional 6% of participants tried to explain, but provided very wrong explanations. 21% declined to reply while 29% wrote they do “not know” or “don’t remember”. 13% knew to explain how the batteries work while 12% tried to but provided inaccurate explanations.

These levels of 33% being able to reproduce at least partially the battery-making process and 13% being able to repeat even more complex scientific or technological explanations show a very promising pilot study for the large scale adaptation of new energy systems through learning. These children were at a summer camp where they are playing and having fun most of the time and experience many workshops and learning activities as they go from one station to another. So they were surrounded with distractions and leisure activities. In these circumstances, it was notable to achieve such levels of attention and actual learning. A future study could evaluate the effect of repeated learning session or a more extended learning program to see if a larger percentage of renewable energy literacy, awareness and perception of personal capabilities is achieved.

Beyond these accounts of personal learning, the children were asked if they think now that Zn/Cu/boiled potato batteries can provide cheap electricity in a simple, easy-to-make way. 54% agreed that yes, it can. 6% said it might be possible but in low volumes, not enough for real consumption. 5% said that it is impossible.

When asked so how can this be achieved, 62% did not reply to this question or said they did not know or did not remember. 31% said they could do it, mostly with the assistance of professional adults, such as a teacher or technician, or with the assistance of parents and family members. The break-down of these replies can be seen in Table 4.

**Table 4.** Do the children think, after the learning session, that they can indeed produce enough energy for lamps at their home using the “make yourself a battery” toolkit and who they think they should do it with. N is the number of children who replied.

Reply	N	%
Declined to answer	34	40
Don’t know, Don’t remember	18	20
Can be done with the help of a professional adult – technician, instructor	10	12
Can be done but we need a lot of potatoes and metal electrodes	10	12
Can be done with the help of parents and family members	6	7
Impossible to do	4	5

Questionnaire Raw Data Kids - Pre Learning Click here for additional data file. Copyright: © 2018 Polikovsky M et al. 2018 Data associated with the article are available under the terms of the Creative Commons Zero "No rights reserved" data waiver (CC0 1.0 Public domain dedication).

Questionnaire Raw Data Kids - Post Learning Click here for additional data file. Copyright: © 2018 Polikovsky M et al. 2018 Data associated with the article are available under the terms of the Creative Commons Zero "No rights reserved" data waiver (CC0 1.0 Public domain dedication).

Raw data for boiled potato batteries performance Click here for additional data file. Copyright: © 2018 Polikovsky M et al. 2018 Data associated with the article are available under the terms of the Creative Commons Zero "No rights reserved" data waiver (CC0 1.0 Public domain dedication).

## Conclusions

Although renewable energy sources are continuously developed, their penetration and adaptation rate by rural population in developing countries is low. These low adaptation rates are associated with unawareness for an alternative to traditional methods solutions and low self-confidence to adopt the new methods. In this study, we developed a toolkit based on Zn/Cu/boiled potatoes batteries and non-formal teaching strategy that could enhance energy literacy and potentially displace the kerosene used for lighting in rural areas. Our pilot study on 96 children from an Israel summer camp, trained by youth leaders, showed that informal renewable energy education increases the awareness and self-confidence for battery assembly and electricity generation at home using simple available materials.

## Ethical statement

The study was conducted according to the research guidelines of the Chief Scientist Office of the Ministry of Education. The educational institution, Teva HaSviva camp, approved of the research plan and informed all parents/guardians about the exact activities that the children would participate in, including interviewing the children, filming and distributing the information online for professional and educational purposes. We received written informed consent from the parents/guardians for the children to participate in the study as a part of their summer camp activities. The research involved only the use of non-sensitive, completely anonymous educational tests, using anonymous questionnaires and interview procedures that did not induce any undue psychological stress or anxiety. This study was done within normal education requirements in Teva HaSviva and to the best of our knowledge is exempt from ethical approval, as per Israel law, which follows Helsinki convention regulation.

## Data availability

The data referenced by this article are under copyright with the following copyright statement: Copyright: © 2018 Polikovsky M et al.

Data associated with the article are available under the terms of the Creative Commons Zero "No rights reserved" data waiver (CC0 1.0 Public domain dedication). 

Dataset 1: Questionnaire Raw Data Kids - Pre Learning. DOI, 10.5256/f1000research.13228.d188989 ^40^

Dataset 2: Questionnaire Raw Data Kids - Post Learning. DOI, 10.5256/f1000research.13228.d188990 ^41^

Dataset 3: Raw data for boiled potato batteries performance. DOI, 10.5256/f1000research.13228.d188991 ^42^

## Supplementary material

> **Supplementary File 1. Teaching materials for renewable energy learning session.**

> **Supplementary File 2. Pre and post learning questionnaires.**

**Table S1. Zn/boiled potato battery characterization table as filled by children.**

**Table S2. Electricity generation competition results.** Measurements and calculations made by the children during competition. Va- voltage on external 100 ohm resistor connected in series with the potato battery. OCV- open circuit voltage.

**Table S3. Pre and post learning questionnaires analysis.**

**Figure S1. Digital images and illustrations of the potato lifecycle during the activity with 96 children.** ( **a**) Two bags of potatoes (about 4 kg each) were used during the activity. ( **b**) The potatoes were cut into pieces. ( **c**) The central part of each potato cut with 1–2 cm intervals, and divided between the central part and the edges. ( **d**) The potatoes were boiled during about an hour. ( **e**) The boiled potato were divided into a mash potato and for slices used the activity purposes ⅓ and ⅔ respectively. ( **f**) All used potatoes after the activity with the children were composted.
