# Supplementary material for: Enhancing energy literacy in children using zn/cu/potato batteries
Source: F1000Res. 2018 Jan 8;7:24. [Version 1] doi: 10.12688/f1000research.13228.1 (PMC6352923; doi:10.12688/f1000research.13228.1)
Supplement: Supplementary file 4 [file f1000research-7-14352-s0003.tgz › d8617a98-473f-429f-abdb-8846791497c7.docx]

**Supplementary File 1**

**Teaching materials for renewable energy learning session**

The main theme of the lesson was led by the question: “How we can learn from the nature to generate renewable energy? By “Nature”, we mean living organisms. The introduction described traditional methods for electricity generation by combusting fossil fuels and its negative impact on the environment. We explained how the combustion reaction causes to a "greenhouse effect” that is thought to increase the global warming by anthropogenic intervention(1).

To answer the question: “How we can learn from Nature to produce renewable energy?”, we provided a few examples from different natural and biologically engineered systems. The model organisms were sunflowers, squid and its symbiotic bacteria, and Zn/boiled potato batteries.

**Example 1 - Sunflowers**. An introduction was given about sunflowers structure and mechanisms for ‘following the sun’, ‘sunflower building structure’ and ‘sunflower solar panel structure’. (a) Follow the sun mechanism: during the day, the young sunflowers can track the sun (circadian clocks) in order to maximize the light absorption (2). Solar panels technology for solar electricity generation was introduced (3). By learning the sunflowers circadian mechanism and implement it on solar panels, the solar panels could improve the energy production by tracking the sun (4). (b) ‘Sunflower building structure’: building with sunflower architecture could improve the building energy consumption (building consume more than 40% of energy in affluent economies). The roof of the building could mimic a flower by including solar panel; thus, similar to roots, the living area would get a natural shadow, reducing total energy requirement for cooling. This sunflower-like building could potentially increase the energy efficiency of the whole building (5). (c) ‘Sunflower solar panel structure’: producing sunflower-like solar panel could be more esthetic than the conservative square structure solar panel, which could make the solar panel more attractive for customers(6).

**Example 2 - Hawaiian bobtail squid.**  *Euprymna scolopes* and its symbiotic luminous bacteria *Vibrio fischeri* (7). A description was given about the squid and an illustration was given about the squid natural predators (fishes). The children gave different guesses how squid protects itself at dark. The answer is the squid has a light organ that allows him to camouflage itself and avoid from predators to detect it at night (8). The bioluminescent bacteria *V. fischeri* released from the light organ(9) at night, and makes the squid be invisible compared to the light that comes from the shallow water (from the moon and stars) (10). This squid-bacterial interaction was introduced to show that there are microorganisms that can produce light (11). If those bioluminescent bacteria would be grown at a proper medium and right conditions, they potentially could be luminescent and be used for human needs such as illumination (12,13).

**Example 3 -**  **production of electricity with boiled potato** (14). In some places around the globe (mainly in developing countries), there is no electricity in the grid (15). In order to get light, the residences at those places have to use expensive and inefficient batteries or kerosene lamps, which could have toxic effects at close places (16). An alternative energetic source was described. A boiled potato that connected to a LED and produces non-toxic cheap light source. Boiling the potato increases efficiency by the reduction in the internal impedance. The power production increased up to 10 times than untreated potato. After the use, the potato can be composted and thus recycled (**Figure S1**).

At the end, the lesson was summarized. The sunflower example was discussed in terms of inspiration from nature; how we can borrow information from it and improve the energy production using the structures that appear in nature. The squid-bacterial interaction was discussed as an example of a way to imply already exist living organisms as a lightning source. The potato example was discussed as a way of electricity production, which using biological source (biomass) and convert energy source for renewable energy production.

**References**

1. Change IP on C. Climate Change 2007 - The Physical Science Basis. Work Gr I Contrib to Fourth Assess Rep IPCC. 2007;1009.

2. Vandenbrink JP, Brown EA, Harmer SL, Blackman BK. Turning heads: The biology of solar tracking in sunflower. Plant Sci. 2014;224:20–6.

3. SolarServer. Photovoltaics: Solar Electricity and Solar Cells in Theory and Practice. SolarServer Global Solar Indusry Website. 2011.

4. RAO BK, RAMESH D, JYOTHI P. Sunflower Model Sunlight Tracking for Solar Panels. 2015;

5. Helen Morgan. Michael Jantzen’s Solar Science Center is Powered by Towering Photovoltaic Antenna. inhabitat. 2012.

6. Dustin Stonecipher. Mueller SunFlowers. AtlasObscura. 2016.

7. Ruby EG. Lessons from a cooperative, bacterial-animal association: the Vibrio fischeri-Euprymna scolopes light organ symbiosis. Annu Rev Microbiol. 1996;50(1):591–624.

8. McFALL-NGAI M, Morin JG. Camouflage by disruptive illumination in leiognathids, a family of shallow-water, bioluminescent fishes. J Exp Biol. 1991;156(1):119–37.

9. Ruby EG, Lee K-H. The Vibrio fischeri-Euprymna scolopes light organ association: current ecological paradigms. Appl Environ Microbiol. 1998;64(3):805–12.

10. Young RE, Roper CF. Bioluminescent countershading in midwater animals: evidence from living squid. Science (80- ). 1976;191(4231):1046–8.

11. Hastings JW, Potrikusv CJ, Gupta SC, Kurfürst M, Makemson JC. Biochemistry and physiology of bioluminescent bacteria. Adv Microb Physiol. 1985;26:235–91.

12. Brad Koerner. Philips microbial home + biolight project. lucept. 2011.

13. Scoles S, Brooke N. SYMBIOSIS: LIGHTING UP THE WORLD. Smaller Questions. 2011.

14. Golberg A, Rabinowitch HD, Rubinsky B. Zn/Cu-vegetative batteries, bioelectrical characterizations, and primary cost analyses. J Renew Sustain Energy. 2010;2(3):1–11.

15. IEA IEA. Energy access database. World Energy Outlook. 2015.

16. Lam NL, Smith KR, Gauthier A, Bates MN. Kerosene: a review of household uses and their hazards in low- and middle-income countries. J Toxicol Environ Health B Crit Rev. 2012;15(6):396–432.
