# Supplementary material for: Enhancing energy literacy in children using zn/cu/potato batteries
Source: F1000Res. 2018 Jan 8;7:24. [Version 1] doi: 10.12688/f1000research.13228.1 (PMC6352923; doi:10.12688/f1000research.13228.1)
Supplement: Supplementary file 5 [file f1000research-7-14352-s0004.tgz › e6249d9e-7652-4372-857b-918330c1ac72.docx]

**Supplementary File 2. Questionnaires.**

**Children - Pre Learning Questionnaire**

How can we create Electricity from Potatoes

Before we start?

All replies are used for the research purpose only

There are no correct or incorrect answers. What you think is whats important

Please mark how much do you agree with each sentence

|  | 5  Very Much | 4  Yes | 3  Sort of | 2  A little | 1  Not at all |
| --- | --- | --- | --- | --- | --- |
| Nature has cool things that are interesting to know about |  |  |  |  |  |
| We can learn from nature very useful things for us |  |  |  |  |  |
| Electricity is there all the time. It never ends |  |  |  |  |  |
| It is possible to create electricity on our own |  |  |  |  |  |
| I can create electricity at my home |  |  |  |  |  |
| I think it will be interesting for me to learn about energy in nature |  |  |  |  |  |
| I think it will be interesting for me to learn to create electricity from potatoes |  |  |  |  |  |
| I am curious to know what will we learn about |  |  |  |  |  |

Please reply to the following questions. If you do not know the answer, no problem. You can just write "don't know" or skip the question

How can we receive or create electricity for our use?

Can we create electricity at home? If yes, how?

How does a battery work? And which types of batteries exist?

What’s the difference between electricity from the power socket or from a battery to electricity in animals or plants?

Can there be any problems or interruptions with electricity supply?

Age: _______

Things I am interested to learn about: _____________________________________

**Children - Post Learning Questionnaire**

How can we create Electricity from Potatoes

Following our study?

All replies are used for the research purpose only

There are no correct or incorrect answers. What you think is what is important

Please mark how much do you agree with each sentence

|  | 5  Very Much | 4  Yes | 3  Sort of | 2  A little | 1  Not at all |
| --- | --- | --- | --- | --- | --- |
| Nature has cool things that are interesting to know about |  |  |  |  |  |
| We can learn from nature very useful things for us |  |  |  |  |  |
| Electricity is there all the time. It never ends |  |  |  |  |  |
| It is possible to create electricity on our own |  |  |  |  |  |
| I can create electricity at my home |  |  |  |  |  |
| It was interesting for me to learn about energy in nature |  |  |  |  |  |
| It was interesting for me to learn how to create electricity from potatoes |  |  |  |  |  |
| I enjoyed this learning session |  |  |  |  |  |
| I can teach others what I learned in this learning session |  |  |  |  |  |

Please reply to the following questions. If you do not know the answer, no problem. You can just write "don't know" or skip the question

In which ways can we receive or create electricity?

Can we create electricity at home? If yes, how?

How does a potato battery work to create electricity?

How do we make a potato battery?

_________________________________

Is it possible to provide electricity from potatoes in a simple and cheap way?______________________________________________________________

______________________________________________________________

If yes, what do need to create enough power for a couple of lamps and who can help us make it?

Age: _______

Things I am interested to learn about: _____________________________________
