# Supplementary material for: Enhancing energy literacy in children using zn/cu/potato batteries
Source: F1000Res. 2018 Jan 8;7:24. [Version 1] doi: 10.12688/f1000research.13228.1 (PMC6352923; doi:10.12688/f1000research.13228.1)
Supplement: Supplementary file 6 [file f1000research-7-14352-s0005.tgz › ed28f1ab-ec86-419a-b382-506e09b62493.docx]

**Table S1. Zn/boiled potato battery characterization table as filled by children.**

| **Name** |  |
| --- | --- |
| **Experiment start time** |  |
| **Group leader name** |  |
| **Number of cells in the battery** |  |
| **OCV (voltage without a load)** |  |
| **Voltage on a 100ohm load** |  |
| **Cell 1 thickness (cm)** |  |
| **Cell 2 thickness (cm)** |  |
| **Cell 3 thickness (cm)** |  |
| **…** |  |
