# Supplementary material for: Enhancing energy literacy in children using zn/cu/potato batteries
Source: F1000Res. 2018 Jan 8;7:24. [Version 1] doi: 10.12688/f1000research.13228.1 (PMC6352923; doi:10.12688/f1000research.13228.1)
Supplement: Supplementary file 7 [file f1000research-7-14352-s0006.tgz › 5f88cc15-29f6-4c1b-9d0e-5a4fe2196656.docx]

**Table S2. Electricity generation competition results.** Measurements and calculations made by the children during competition. Va- voltage on external 100 ohm resistor connected in series with the potato battery. OCV- open circuit voltage.

| **Power density [Watt/cm^2^ ]** | **Current density [A/cm^2^ ]** | **Power [Watt]** | **Current [A]** | **Std of working electrode surface [cm]** | **Working electrode surface[cm]** | **OCV [Volt]** | **Va [Volt]** | **Thickness average (cm)** | **Num. batteries in each assembly** | **Batteries assembly num.** |
| --- | --- | --- | --- | --- | --- | --- | --- | --- | --- | --- |
| 8.4E-06 | 5.3E-05 | 2.6E-04 | 1.6E-03 | 4.40 | 30.39 | 2.85 | 0.16 | 1.05 | 4 | **1** |
| 8.0E-06 | 5.0E-05 | 2.6E-04 | 1.6E-03 | 7.61 | 32.11 | 9.77 | 0.16 | 0.94 | 15 | **2** |
| 2.9E-06 | 2.9E-05 | 1.0E-04 | 1.0E-03 | 5.48 | 33.90 | 5.37 | 0.10 | 1.26 | 10 | **3** |
| 1.8E-05 | 7.0E-05 | 6.8E-04 | 2.6E-03 | 4.67 | 37.33 | 5.92 | 0.26 | 1.63 | 8 | **4** |
| 1.8E-05 | 7.0E-05 | 6.8E-04 | 2.6E-03 | 6.32 | 37.29 | 6.07 | 0.26 | 1.22 | 9 | **5** |
| 4.0E-06 | 3.3E-05 | 1.4E-04 | 1.2E-03 | 4.16 | 35.83 | 5.59 | 0.12 | 1.64 | 8 | **6** |
| 2.2E-05 | 7.7E-05 | 7.8E-04 | 2.8E-03 | 5.84 | 36.20 | 11.70 | 0.28 | 1.26 | 16 | **7** |
| 1.2E-05 | 6.4E-05 | 3.6E-04 | 1.9E-03 | 7.43 | 29.59 | 3.64 | 0.19 | 1.22 | 5 | **8** |
| 7.5E-06 | 4.4E-05 | 2.9E-04 | 1.7E-03 | 6.63 | 38.35 | 8.05 | 0.17 | 1.30 | 11 | **9** |
| 1.7E-05 | 6.9E-05 | 6.3E-04 | 2.5E-03 | 5.77 | 36.09 | 8.90 | 0.25 | 1.17 | 14 | **10** |
| 1.9E-05 | 7.5E-05 | 6.3E-04 | 2.5E-03 | 6.01 | 33.15 | 3.81 | 0.25 | 1.12 | 13 | **11** |
|  |  | 6.8E-04 | 2.6E-03 |  |  | 13.65 | 0.26 | 1.20 | 20 | **12** |
|  |  | 7.3E-04 | 2.7E-03 |  |  | 15.30 | 0.27 | 1.25 | 21 | **13** |
|  |  | 3.2E-04 | 1.8E-03 |  |  | 12.35 | 0.18 | 1.18 | 18 | **14** |
|  |  | 4.0E-04 | 2.0E-03 |  |  | 8.23 | 0.20 | 1.04 | 12 | **15** |
|  |  | 2.9E-04 | 1.7E-03 |  |  | 15.38 | 0.17 | 1.27 | 21 | **16** |
|  |  | 2.9E-04 | 1.7E-03 |  |  | 16.35 | 0.17 | 1.15 | 22 | **17** |
|  |  | 2.3E-04 | 1.5E-03 |  |  | 7.98 | 0.15 | 1.29 | 11 | **18** |
|  |  | 3.2E-04 | 1.8E-03 |  |  | 4.30 | 0.18 | 1.12 | 6 | **19** |
|  |  | 2.9E-04 | 1.7E-03 |  |  | 16.55 | 0.17 | 1.10 | 23 | **20** |
|  |  | 1.0E-04 | 1.0E-03 |  |  | 7.03 | 0.10 | 1.50 | 12 | **21** |
|  |  | 3.2E-04 | 1.8E-03 |  |  | 9.04 | 0.18 | 1.63 | 12 | **22** |
