# Supplementary figures and images for: Enhancing energy literacy in children using zn/cu/potato batteries
Source: F1000Res. 2018 Jan 8;7:24. [Version 1] doi: 10.12688/f1000research.13228.1 (PMC6352923; doi:10.12688/f1000research.13228.1)

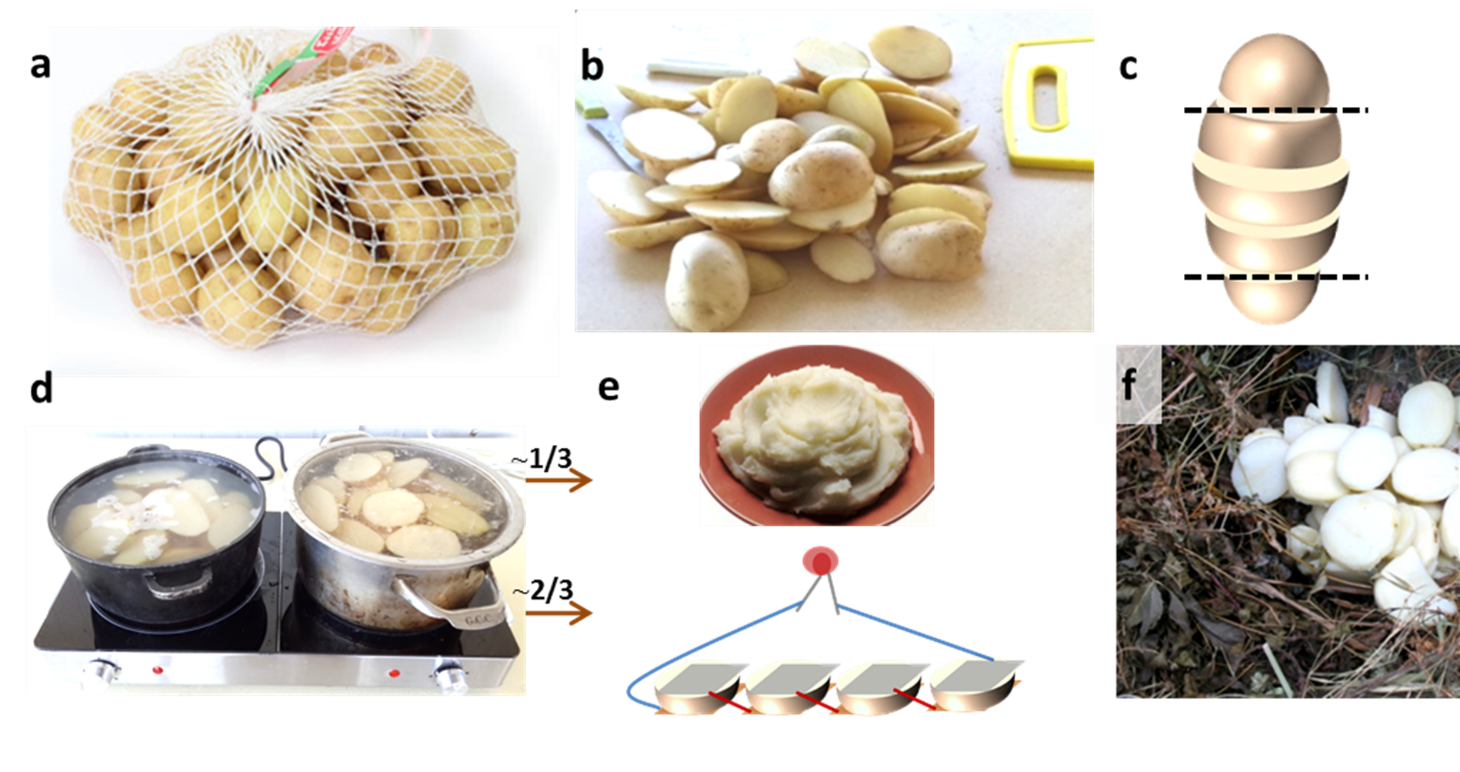

Supplement: Supplementary file 9 [file f1000research-7-14352-s0008.tgz › 0de7bfd1-c473-445b-8fad-4e49153c33f8.png]
