# Supplementary material for: Enhancing energy literacy in children using zn/cu/potato batteries
Source: F1000Res. 2018 Jan 8;7:24. [Version 1] doi: 10.12688/f1000research.13228.1 (PMC6352923; doi:10.12688/f1000research.13228.1)
Supplement: Peer review history

# Peer review: Referee response for version 1

**DOI:** 10.5256/f1000research.14352.r43040

**Published:** 2019-02-11

**Authors:** Sue Wake

**Affiliations:** 1 Department of Architecture, Unitec Institute of Technology, Auckland, New Zealand

Your paper is interesting and relevant, but there are a number of areas that should be attended to, in order to strengthen its content readability and therefore its research value. I outline these here, in the order of reading:

 1.  You should state upfront that this is a pilot study and you are using child participants from affluent backgrounds, with the assumption that the results should be more valuable if applied to children from poorer backgrounds. You should provide some evidence of why you have this assumption - e.g. academic studies. Currently it is confusing as you introduce the idea of the pilot study further into the paper, and, even later, you admit that your subjects are from wealthy backgrounds and you recommend repeating the project with poorer children, since this could/should yield more positive results.

 2.  In your introduction you should more obviously credit the 2 models in Figs 1a and b.  The way it is currently presented makes them seem like your own.

 3.  The paragraph in your intro starting 'Previous works...' is inelegantly written and not well linked to the topic of this paper. I recommend you reduce its length and more strongly connect it to your project in terms of your chosen focus (i.e. informal outside education).

 4.  It feels that there is a lost opportunity in this paper with regards making more of the youth leader role.  This is an excellent example of 'peer learning' and reference to the literature on this (under children's participation) would give greater depth to your research and also extend the learning potential of your project.  An example reference is Harry Shier's 'Pathways to participation' revisited: Learning from Nicaragua's child coffee workers ^1^. 

 5.  The second to last para of your intro, beginning 'At the same time ... the cost of light...'  What do you mean by the cost of light? You then write 'This study was followed...' This sentence needs clarifying and more detail provided. For example, you might say 'A similar study from Sri Lanka found ... due to lack of awareness and poor self-confidence'. The word 'populations' should have the 's' removed. 

 6.  In general you don't have enough references to the qualitative aspects of your research. More background on this will strengthen your argument considerably.

 7.  Your sections on 'Battery Performance Evaluation' and 'Electrical Production' sit very awkwardly in relation to the context of your research. I question what is the relevance of these sections? If it is important to your paper to produce these figures, then you need to put them into context and explain why they are relevant to your paper.

 8.  The information about the youth guides on P.5 is a little ambiguous as to whether the whole section just refers to them, or also to the other child participants. Perhaps give this a section heading e.g. 'Youth Guide Training'.

 9.  Also P.5 - I think you mean 'fertiliser', not 'fertilisation'.

 10.  'Results & Discussion' - the sentence about the longest battery should be changed to 'longest battery assembled by children' - to make sense.

 11.  There are a number of places where the English is not quite correct and the whole paper needs close editing for this. For example, where 'can' should be replaced by 'could'. 

 12.  Is Fig 4 really of value to your paper? I am not convinced it is.

 Overall, while your paper about the pilot study has some valuable outcomes, there are some significant issues with the number of assumptions you make, without evidence to support your rationale. Strong evidence of the benefits to children's education about alternative power sources and the viability of this method as a way of generating bio-energy (e.g. a whole lot of cooked potatoes presents a space and hygiene problem) seem to be rather absent from your paper. The observation that the 41% of participants that mentioned potatoes as an energy source, probably guessed this answer from inferences already made to them, sort of sums up the inconclusiveness of your results. This is reflected in your very brief conclusion. The paper needs to gain more substance through reducing some of the current content (as suggested) to allow space to focus more strongly and clearly on the background to children's learning by this method, linked through to your results. Currently there are too many loose ends. I also recommend that if you do any follow-up research, that you include the voice of the youth leaders and questions asked of the child and youth participants about how this way of learning compared to learning from adults.

I have read this submission. I believe that I have an appropriate level of expertise to confirm that it is of an acceptable scientific standard, however I have significant reservations, as outlined above.

## References

[1] ShierH: Pathways to participation’ revisited: Learning from Nicaragua’s child coffee workers. *A Handbook of Children’s Participation: Perspectives from Theory and Practice*.2009;215-227

# Peer review: Referee response for version 1

**DOI:** 10.5256/f1000research.14352.r42920

**Published:** 2019-01-28

**Authors:** Ikerne Aguirre Bielschowsky

**Affiliations:** 1 School of Geography, University of Melbourne, Melbourne, VIC, Australia

I found this article interesting, useful for educators, well written and structured, and easy to follow. All of the raw data are made available, and the paper provides enough information to replicate the activity. The ideas presented here are innovative, and could have practical implications when applied in a low-income, off-the-grid context – indeed, I very much look forward to the follow-up study focusing on this topic. Before moving on, however, there are a few points that need to be clarified.

**Major comments**

1. Overall, I think that the framing of the study needs to be more explicit on what the authors wish to achieve and, crucially, what they hope to achieve here vs in a future study building on the current results. The abstract and introduction suggest a focus on developing the children’s awareness of energy sources and their impact on society and the environment, as well as changing their energy behaviour. Yet the project itself mostly deals with the practical and scientific side of making electricity from potatoes. If the children were involved in discussions on (the impact of) current energy sources, and/or what difference using potatoes could actually make, please elaborate on what specific topics were discussed, how they were discussed, and the final understanding obtained by the children. I think the paper ought to be clear throughout on what are the secondary aims (apart from building self-confidence) E.g. to (i) increase children’s scientific knowledge on the process of electricity production; (ii) serve as a pilot study for a bigger research programme, which intends to improve living conditions in off-grid areas; and/or (iii) develop children’s energy literacy. All three are worthwhile, and indeed all of them are mentioned here; however, not all of them are properly discussed, especially the energy literacy part. For example, the children are seemingly meant to realise the connection between generating electricity from potatoes and the wider socio-environmental implications of energy consumption and production. Understanding this complicated relationship likely would require different or additional learning experiences and follow-up evaluations.
2. According to the results (Table 2), most children think that electricity in general is generated from potatoes (both before and after the activity). This statement somewhat worries me, as it seems to reveal a misunderstanding about how electricity is actually produced in our society. Is there any indication that the children understand the broader context?
3. It would be helpful to have an idea of the characteristics of the participating children. How many boys vs girls participated? Are they mostly the children of academics, or do they come from underprivileged neighborhoods? What is their general socioeconomic background? Also, there ought to be some information on their previous experience. Is energy education compulsory in primary schools in Israel? Would the children have had previous experience doing experiments involving electricity, conductivity, etc.?

**Minor comments**

1. It is important to acknowledge in the introduction (e.g. bottom of p.2) that energy-related behaviours depend on external factors as well as internal/psychological ones, e.g. material culture and social norm. The latter are especially important when working with children.
2. Educators thinking of running this project with their students might benefit from an idea of how much a complete kit costs. 
3. I think you mean technology “adoption”, rather than “adaptation”.
4. The section on “Qualitative assessment of knowledge and attitude” is somewhat repetitive, as it mostly recalls information already presented in the tables. I think it could be significantly shortened by discussing only the main trends. Also, I wonder whether the qualitative aspect of this section could be enhanced via some quotes, and a more in-depth discussion of the participants’ opinions.

I have read this submission. I believe that I have an appropriate level of expertise to confirm that it is of an acceptable scientific standard.

# Peer review: Referee response for version 1

**DOI:** 10.5256/f1000research.14352.r29584

**Published:** 2018-05-14

**Authors:** Srikanth Mutnuri

**Affiliations:** 1 Department of Biological Sciences, Birla Institute of Technology and Science, Pilani (BITS Pilani), Zuarinagar, India

The paper is well written. However I am not sure how the potato experiment can increase the awareness of children about clean energy possibilities, and increase their self-confidence in applying them. This experiment can certainly give knowledge about how electricity can be generated. I am not sure about it as clean energy possibilities. I feel this can be highlighted in the paper.

I have read this submission. I believe that I have an appropriate level of expertise to confirm that it is of an acceptable scientific standard.
